# Prognostic Value of Preoperative Pro-B-Type Natriuretic Peptide: Early Predictor of Cardiovascular Complications and Mortality After Major Abdominal Surgery

**DOI:** 10.7759/cureus.11338

**Published:** 2020-11-05

**Authors:** Muhammad Nadeem Khurshaidi, Asad Waqar, Muhammad Sohaib Asghar, Afshan Kiran, Asma Tariq, Noureen Kanwal, Rumael Jawed, Uzma Rasheed, Maira Hassan, Anees Mazhar

**Affiliations:** 1 General Surgery, Liaquat National Hospital, Karachi, PAK; 2 Internal Medicine, Dow University of Health Sciences, Karachi, PAK; 3 Internal Medicine, Shaheed Mohtarma Benazir Bhutto Medical College, Lyari General Hospital, Karachi, PAK; 4 Internal Medicine, Liaquat National Hospital, Karachi, PAK; 5 Orthopaedics, Liaquat National Hospital, Karachi, PAK

**Keywords:** surgery, cardiovascular, n terminal pro bnp, cardiac bnp, cardiac failure, myocardial infarction, post operative complication, survival analysis, predictability, outcome

## Abstract

Background and objectives

In surgical patients, coronary disease is the main cause of perioperative mortality. The incidence of serious cardiovascular complications is reported as 5% with a probability of 1-2% of death from the cardiac cause in major non-cardiac surgery. B-type natriuretic peptide (BNP) is a sensitive and specific predictor of left ventricular systolic dysfunction and predicts first cardiovascular event and death in the general population. The recent guidelines recommended the use of pro-BNP for independent perioperative prognosis in cardiac patients undergoing non-cardiac surgery. The aim of this study is to assess the predictive value of raised pro-BNP levels in patients who underwent major abdominal surgery and evaluate its relationship with cardiovascular complications and mortality occurring up to 30 days after surgery.

Materials and methods

We reviewed the medical records of patients undergone surgical procedures in the abdominal region lasting more than two hours, requiring postoperative high dependence or intensive care and an expected hospital stay of at least three days. All types of open or laparoscopic-assisted abdominal or pelvic surgeries that were evaluated for preoperative pro-BNP levels were included in the study. During the postoperative period, all patients were followed for cardiac complications and mortality for 30 days after surgery. Postoperative adverse cardiac events were predefined as angina pectoris, myocardial infarction, cardiogenic dyspnea, acute arrhythmias (atrial fibrillation/flutter, ventricular fibrillation/tachycardia), acute hypertensive event (hypertensive emergency or urgency), congestive heart failure, acute pulmonary edema, or primary cardiac death. While non-cardiac complications were also documented as either pulmonary, septic, postsurgical site infection, and other systemic complications. Subsequently, a survival analysis was done for the discretion of cardiovascular complications and mortality.

Results

The mean age of the study population was found to be 50.22 ± 14.28 years, mean pro-BNP levels were 909.29 ± 3950.04, and mean days of hospital stay were 7.43 ± 4.49 days. The 30-day postoperative all-cause mortality was found to be 9.8%. Hypertension and diabetes were frequent comorbidities amongst the study population. The mean preoperative pro-BNP levels were found higher in the male gender (p=0.071), also found higher in those with cardiovascular complications (p=0.006) and mortality (p=0.057). Receiver operating characteristic (ROC) analysis showed cardiovascular outcomes with a cut-off value of pro-BNP at 143 pg/ml, AUC of 0.891, at a sensitivity of 91%, positive predictive value (PPV) of 96%, a specificity of 75%, and negative predictive value (NPV) of 58%, while the same for mortality at a cut-off value of 164 pg/ml was found with AUC of 0.815, at a sensitivity of 84%, a specificity of 66%, PPV of 97%, and NPV of 21%. The unadjusted odds ratio for cardiovascular complications was found to be 17.857 (95% CI: 6.56-48.60) while that for mortality was 10.863 (95% Cl: 2.29-51.37). The Kaplan-Meier survival curves showing elevated pro-BNP levels were significantly associated with cardiovascular events, with 30 days mortality at a cut-off value of 164 pg/ml.

Conclusion

Pro-BNP is a useful marker in postoperative patients for not only predicting cardiovascular outcomes as cited by many previous studies but also mortality.

## Introduction

In surgical patients, coronary disease is the main cause of perioperative mortality. Every year 100 million patients worldwide undergo non-cardiac surgery out of which 500,000-900,000 patients develop perioperative adverse cardiac events [1]. The incidence of serious cardiovascular complications is reported as 5% with a probability of 1-2% of death from the cardiac cause in major non-cardiac surgery [2]. Several indexes and many other scoring systems have been postulated to predict postoperative adverse cardiovascular events. The American College of Cardiology/American Heart Association guidelines, American Society of Anesthesiologists grading, Goldman cardiac risk index, the modified revised cardiac index, APACHE II Acute Physiology, and myocardial stress tests were advised for preoperative risk stratification in surgical patients, but each method has some limitation in providing useful clinical information about the perioperative risk, has limited prognostic value, limited overall accuracy, and is not commonly applied in everyday practice [1].

B-type natriuretic peptide (BNP) is a sensitive and specific predictor of left ventricular systolic dysfunction and predicts first cardiovascular event and death in the general population [1]. BNP >400 pg/ml is a diagnostic indicator of cardiac breathlessness and is a prognostic and diagnostic marker of heart failure and acute coronary syndromes. BNP also has a prognostic role in ischemic heart events, and BNP levels have been shown to predict both death and cardiovascular events. Recent research suggests that preoperative BNP levels are predictive of postoperative cardiac complications in patients following coronary artery bypass grafting [1-3]. Both BNP and N-terminal pro-B-type natriuretic peptide (NT-proBNP) are derived from pro-BNP. BNP is a 32-amino-acid peptide secreted primarily by ventricular myocytes and fibroblasts in response to ventricular filling pressures and increased wall stress induced by volume expansion, pressure overload, or ischemia of the cardiac chambers. BNP also decreases blood pressure and increases vasodilation. BNP is degraded by endopeptidases with a half-life of 5-10 min [3,4].

The recent guideline from the European Society of Cardiology (ESC) and the European Society of Anesthesiology (ESA) recommended using pro-BNP for independent perioperative prognosis in high-risk cardiac patients undergoing non-cardiac surgery [5]. In a recent prospective cohort study, 31 of 205 (15%) patients had adverse cardiac events in the postoperative period up to 30 days after discharge [6]. About five patients (2.4%) of these 31 died from cardiac events. Preoperative BNP values were significantly increased in the 31 patients with adverse cardiac events compared to the patients without adverse cardiac events in the postoperative period (72 hours after surgical procedure). Furthermore, postoperative BNP values correlate with postoperative adverse cardiac events. Assessing the utility of preoperative BNP as a predictor of postoperative adverse cardiac events shows a sensitivity of 80.6%, and a specificity of 67.2% as compared to a sensitivity of 51.6% and a specificity of 55.7% of revised cardiac risk index values >1 [1]. Patients identified as high risk may require a more detailed preoperative cardiac evaluation and perioperative optimization. There is a lack of objectivity in this approach, and essential cardiovascular complications could be overlooked. Therefore, there is a need for a reliable objective screening test to risk-stratify patients and to identify those requiring a more detailed preoperative investigation. The importance of BNP in cardiac diseases and early data suggest that BNP could find a role in the perioperative period as a stratification marker of increased risk of postoperative cardiac events [7].

The utility of BNP in risk stratification of patients undergoing major abdominal surgery has not been previously tested. With pro-BNP established as clinically relevant, efforts should be made to determine whether preoperative pro-BNP being an economical marker can be used to improve the risk stratification of surgical patients and may have utility in predicting the outcome of major abdominal surgery. The fact that the majority of major postoperative complications are cardiovascular and can be avoided prompts this interest. The aim of this study is to assess the predictive value of raised pro-BNP levels as a risk factor in patients who underwent major abdominal surgery and evaluate its relationship with cardiovascular complications and mortality occurring up to 30 days after surgery in a general surgery department of a tertiary care hospital.

## Materials and methods

We reviewed the medical records of patients undergone surgical procedures in the abdominal region lasting more than two hours, requiring postoperative high dependence or intensive care and an expected hospital stay of at least three days. All the patients included were falling in the American Society of Anesthesiologists (ASA) classification of physical status 1-4. The study excluded all those patients with minor or intermediate open and laparoscopic surgery, such as cholecystectomy, appendectomy, inguinal hernia repair, closure of ileostomy/colostomy, gynecological, and urological procedures. Apart from that, all patients with valvular heart disease, those receiving hemodialysis or peritoneal dialysis for renal failure, and those with ASA physical status 5 (such patients are not expected to survive with or without surgery, and their underlying illness is expected to have an overwhelming effect on the outcome) were excluded from the sample. The data were obtained from a single-center, tertiary care hospital between February 2020 and September 2020, after the ethical approval was waived by the institutional review board. Written consent was obtained from the relevant head of department before undergoing data collection. All types of open or laparoscopic-assisted abdominal or pelvic surgeries including gastrointestinal operations (colorectal, gastric, small bowel, pancreatic, and hepatobiliary surgery, esophagectomy, abdominal incisional hernia repair with or without bowel resection) that were evaluated for preoperative pro-BNP levels were included in the study. During the postoperative period, all patients were followed for cardiac complications and mortality for 30 days after surgery. Postoperative adverse cardiac events were predefined as angina pectoris, myocardial infarction, cardiogenic dyspnea, acute arrhythmias (atrial fibrillation/flutter, ventricular fibrillation/tachycardia), acute hypertensive event (hypertensive emergency or urgency), congestive heart failure, acute pulmonary edema, or primary cardiac death. While non-cardiac complications were also documented as either pulmonary, septic, postsurgical site infection, and other systemic complications.

The sample size is calculated using the World Health Organization (WHO) sample size calculator considering the prevalence of patients having adverse cardiovascular events predicted by pro-BNP, P = 15% taking confidence level 95% and margin of error = 7.5%. The total sample size came out to be 132 patients. A non-probability consecutive sampling technique was used. The patient’s data were compiled and analyzed through the Statistical Package for Social Sciences (IBM SPSS, Version 25, IBM Corp., Armonk, USA). Subsequently, a survival analysis was done to discrete cardiovascular outcomes and mortality. Frequencies and percentages were computed for qualitative variables like gender, diagnosis, surgical procedure, cardiovascular complications, and mortality. Means and standard deviations were described for quantitative data. A multinomial logistic regression model was used for univariate regression analysis and an unadjusted odds ratio (OR) was obtained with a 95% confidence interval. A receiver operating characteristic (ROC) analysis was adopted for the cut-off prediction of pro-BNP values with cardiovascular complications and mortality by obtaining area under the curve (AUC). Kaplan-Meier survival curves were generated for the variables with a log-rank (Mantle-cox) chi-square values. P ≤ 0.05 will be considered significant.

## Results

The mean age of the study population was found to be 50.22 ± 14.28 years, the mean pro-BNP levels were 909.29 ± 3950.04, and mean days of hospital stay were 7.43 ± 4.49 days. The 30-day postoperative all-cause mortality was found to be 9.8%. Hypertension and diabetes were frequent comorbidities amongst the study population. The major abdominal surgeries performed along with their indications are slated in Table 1.

**Table 1:** Baseline characteristics of the study population (n=132). Data are presented as either mean ± standard deviation or frequency and percentage: n(%). NT-proBNP: N-terminal pro-B-type natriuretic peptide, DM: diabetes mellitus; HTN: hypertension; IHD: ischemic heart disease; COPD: chronic obstructive pulmonary disease, CA: carcinoma, CBD: common bile duct, GIST: gastrointestinal stromal tumor, APR: abdominoperineal resection.

Mean age		50.22 ± 14.28	
Mean pro-BNP levels		909.29 ± 3950.04	
Mean days of stay		7.43 ± 4.49	
Mortality		13/132 (9.8%)	
Gender			
Males		57 (43.2%)	
Females		75 (56.8%)	
Comorbidities			
DM		39 (29.5%)	
HTN		48 (36.4%)	
IHD		9 (6.8%)	
COPD		6 (4.5%)	
Others		19 (14.4%)	
Malignancy		46 (34.8%)	
No-comorbidities		60 (45.4%)	
Reason for surgery			
Abdominal mass		4 (3.0%)	
Acute cholecystitis		7 (5.3%)	
CA esophagus		8 (6.0%)	
CA pancreas		6 (4.5%)	
CA rectosigmoid colon		6 (4.5%)	
CA rectum		8 (6.0%)	
CA sigmoid colon		9 (6.8%)	
CBD injury		5 (3.7%)	
Choledocholithiasis		7 (5.3%)	
Duodenal mass		2 (1.5%)	
Evisceration of small bowel		3 (2.3%)	
GIST		3 (2.3%)	
Hirschsprung's disease		2 (1.5%)	
Incisional hernia		16 (12.1%)	
Large bowel obstruction		7 (5.3%)	
Small bowel obstruction		11 (8.3%)	
Obstructed hernia		7 (5.3%)	
Pneumoperitoneum		9 (6.8%)	
Symptomatic gallstones		7 (5.3%)	
Pyloric obstruction		5 (3.7%)	
Surgical procedure performed			
Two-stage esophagectomy		5 (3.7%)	
Exploratory laparotomy + APR		9 (6.8%)	
Exploratory laparotomy + low anterior resection		8 (6.0%)	
Exploratory laparotomy + prepyloric perforation repair		9 (6.8%)	
Feeding gastrostomy		3 (2.3%)	
Feeding jejunostomy		1 (0.7%)	
Gastrojejunostomy		3 (2.3%)	
Incisional hernia mesh repair		10 (7.5%)	
Laparotomy + adhesiolysis		18 (13.6%)	
Laparotomy + small bowel resection and anastomosis		2 (1.5%)	
Laparotomy + sigmoid colectomy		6 (4.5%)	
Laparotomy + Choledochojejunostomy		5 (3.7%)	
Laparotomy + excision of mass		4 (3.0%)	
Laparotomy + Hartmann’s reversal		4 (3.0%)	
Laparoscopic sigmoid colectomy		5 (3.7%)	
Laparotomy + Vicryl mesh laparostomy		3 (2.3%)	
Obstructed hernia mesh repair		7 (5.3%)	
Incisional hernia mesh repair + abdominoplasty		6 (4.5%)	
Open cholecystectomy		8 (6.0%)	
Open cholecystectomy + CBD exploration		11 (8.3%)	
Whipple’s procedure		5 (3.7%)	

**Figure 1:**
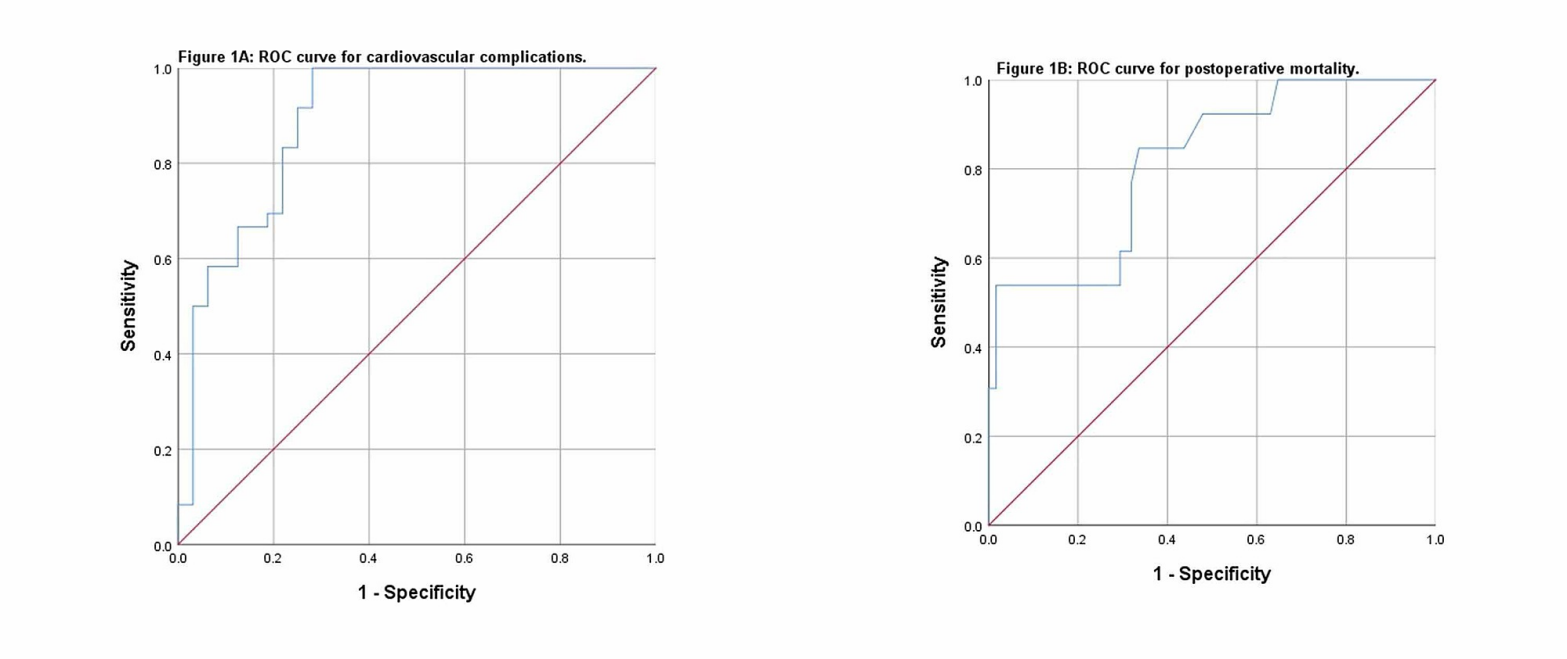
ROC statistics for cardiovascular complications (A) and mortality (B). ROC: receiver operating characteristic.

The mean preoperative pro-BNP levels were found higher in the male gender (p=0.071), also found higher in those with cardiovascular complications (p=0.006) and mortality (p=0.057). ROC analysis showed cardiovascular outcomes with a cut-off value of pro-BNP at 143 pg/ml, AUC of 0.891 (95% CI: 0.83-0.94) at a sensitivity of 91%, positive predictive value (PPV) of 96%, a specificity of 75%, and negative predictive value (NPV) of 58%, as shown in Table 2 and Figure 1(A).

**Table 2:** Descriptive and inferential statistics for pro-BNP as a predictor of cardiac event and mortality. NT-proBNP: N-terminal pro-B-type natriuretic peptide, AUC: area under the curve, 95% CI: 95% confidence interval, SE: standard error, PPV: positive predictive value, B: unstandardized beta, OR: odds ratio, df: degrees of freedom, ROC: receiver operating characteristic.

Postoperative complications	Cardiac		Non-cardiac		None	
	n=36 (27.2%)		n=51 (38.6%)		n=45 (34.0%)	
Mean pro-BNP levels	2657.88 ± 7207.96		396.16 ± 1197.31		91.97 ± 84.85	
ROC analysis						
Outcome variable	AUC:	95% CI	SE	Sensitivity	PPV	p-value
Cardiac event (cut-off for pro-BNP: 143.0 pg/ml)	0.891	0.83–0.94	0.027	91.7%	96.0%	<0.001
Mortality (cut-off for pro-BNP: 164.0 pg/ml)	0.815	0.69–0.93	0.061	84.6%	97.5%	<0.001
Univariate logistic regression analysis						
Cardiac event	B: 2.882	OR: 17.857	Wald: 31.838	95% CI: 6.56–48.60	SE: 0.285	p-value: <0.001
Mortality	B: 2.385	OR: 10.863	Wald: 9.052	95% CI: 2.29–51.37	SE: 0.340	p-value: 0.003
Kaplan-Meier survival analysis						
For cardiac event	Log-rank (Mantel-cox): 5.507			df: 2	p-value: 0.064	
For mortality	Log-rank (Mantel-cox): 4.250			df: 1	p-value: 0.039	

**Figure 2:**
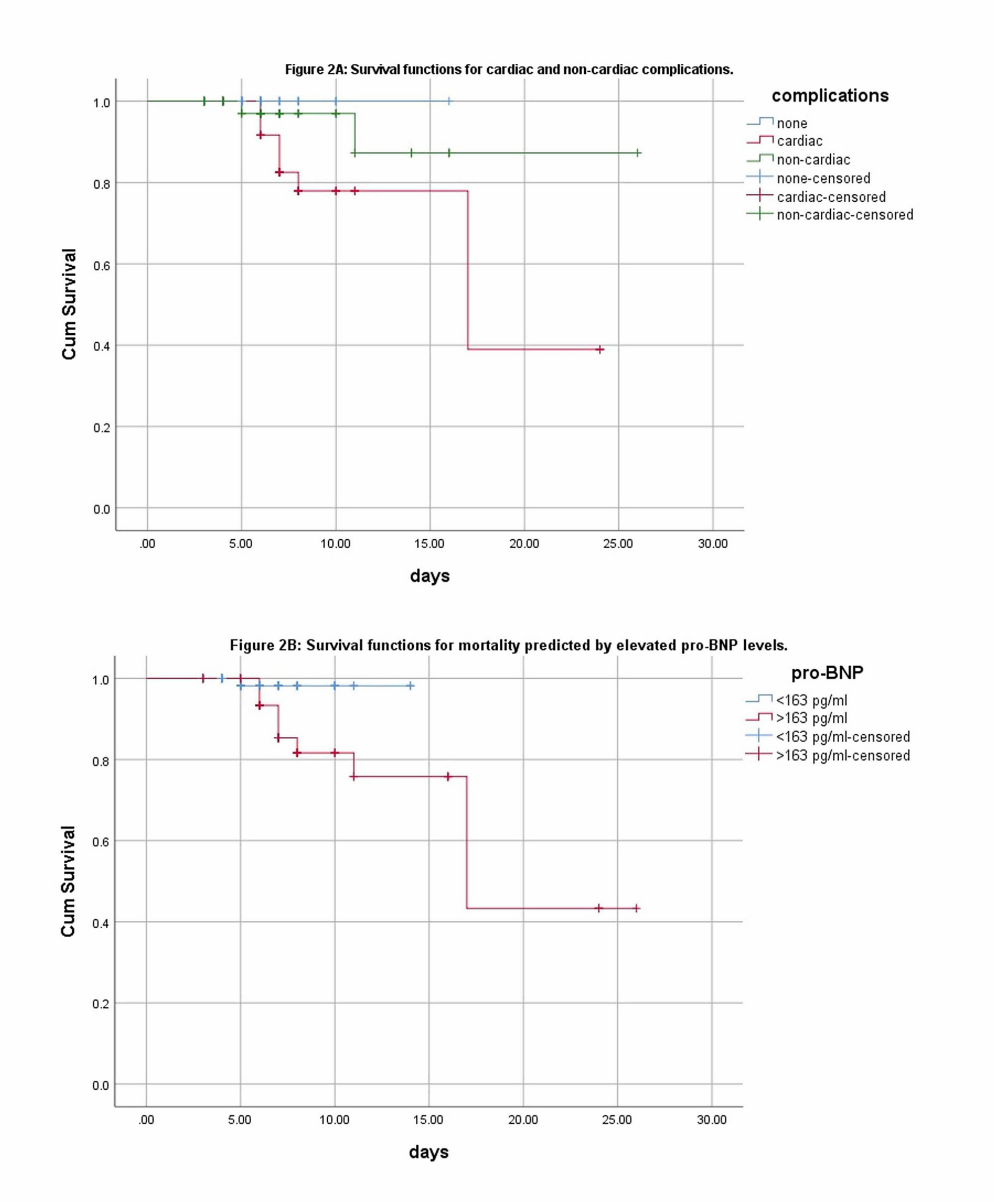
Kaplan-Meier curves for cardiovascular complications (A) and mortality (B). BNP: B-type natriuretic peptide.

The same for mortality at a cut-off value of 164 pg/ml was found with AUC of 0.815 (95% CI: 0.69-0.93), at a sensitivity of 84%, a specificity of 66%, PPV of 97%, and NPV of 21%, as shown in Figure 1(B). The unadjusted odds ratio for cardiovascular complications was found to be 17.857 (95% CI: 6.56-48.60) while that for mortality was 10.863 (95% CI: 2.29-51.37), both statistically significant. The Kaplan-Meier survival curves showing elevated pro-BNP levels were significantly associated with cardiovascular events as shown in Figure 2(A), with 30 days mortality at a cut-off value of 164 pg/ml (Figure 2(B)).

## Discussion

The mean age of patients suffering from cardiovascular complications and mortality after major cardiac and non- cardiac surgeries is ranging from 72 years to 77 years in multiple studies [1,2,6,8-10]. A study conducted by Fox et al. quoted the mean age of 63 years contradicting outcomes of miscellaneous studies [9]. The male gender was significantly affected when compared with the female gender in numerous trials conducted [1,2,6,8,10]. Fox et al reported female gender more affected thus contravening abundant studies [9]. The most prevalent co-morbidity encountered among patients was hypertension with 70-77% [5,8] followed by diabetes mellitus with 29-48% [1,2,6,8-10]. The least prominent co-morbidities were coronary artery disease with 29% [1] and congestive heart failure with 10.9% [8]. Major adverse cardiovascular events (MACE) such as preoperative complications include non-fatal myocardial infarction with 87% followed by heart failure 71%, atrial hypertension 58%, cardiac arrests 43%, ischemic heart disease 40%, atrial fibrillation 34%, peripheral arterial disease 33%, non-fatal cardiac arrests 28% while infrequent adverse events were dilated cardiomyopathy with 12% and ventricular fibrillation 6% [1,2,6,10]. Around 42.8% of patients underwent abdominal surgery in one of the studies [5]. Measurement levels of pro-BNP was an independent factor in predicting prognosis and mortality due to cardiac events among patients undergoing non-cardiac surgeries. Increased levels of pro-BNP were detected among patients suffering from adverse cardiac events when compared to patients with no cardiac events [1,6,9,11]. The mortality percentile among patients with increased levels of preoperative pro-BNP levels was reported 7.8% in a study regulated by Zurro et al. [2] while the least percentile of mortality was reported as 3.3% by Gregg et al. [10].

A meta-analysis conducted to find out the association of elevated preoperative pro-BNP showed significant cardiovascular complications like myocardial infarction, atrial fibrillation, and sudden cardiac death, with an odds ratio of 19.3 which was comparable with our odds ratio for a cardiac event of 17.8 [11]. Similarly, the odds ratio for mortality in the discussed study was 14.7, which was slightly higher than our finding of 10.8, both statistically significant [12]. Another study predicted ventricular dysfunction at an odds ratio of 1.92 while mortality at 1.89 with elevated preoperative pro-BNP levels [9]. The incidence of cardiovascular complications was found to be 7.8% in one study [2], which was much lesser when compared to our study population (27.2%) and we also reported a mortality rate of 9.8%. However, the study indicated significant associations of preoperative pro-BNP elevation with cardiovascular complications (OR: 8.7-22.0) and mortality (6.2-23.8) comparable with our findings [2]. One such study also claimed 9.96% postoperative cardiac events and associated preoperative pro-BNP elevation at an odds ratio of 4.81 with Kaplan-Meier survival curves comparable to our findings [6]. Another meta-analysis showed significant associations of pro-BNP with postoperative cardiac events (OR: 19.77) and mortality (OR: 9.28), similar to our findings [7]. While such associations are expected and already proven previously in cardiac-related surgeries [8,9], its prognostic value in non-cardiac surgery has been gaining significance recently.

A study showed elevated preoperative pro-BNP levels predicting mortality and cardiovascular outcomes at a sensitivity of 75% and specificity of 70% with an AUC of 0.72 and a cut-off value of 40 pg/ml (OR: 6.76) [11], while in our study, we predicted cardiovascular outcomes with a cut-off value 143 pg/ml, AUC of 0.89 at a sensitivity of 91%, PPV of 96%, a specificity of 75%, and NPV of 58%. The same for mortality in our study was at a cut-off value of 164 pg/ml, AUC of 0.81, 84% sensitivity, 66% specificity, 97% PPV, and 21% NPV. Another study predicted utility of preoperative pro-BNP for cardiovascular events at a cut-off value 36 pg/ml with AUC of 0.778, 80.6% sensitivity and 67.2% specificity (OR: 4.64) [1]. One such study also associated postoperative pro-BNP levels with mortality and cardiovascular events and giving similar results [8].

## Conclusions

As we know that major abdominal surgeries are a risk factor for increased cardiac load and postoperative cardiac complications. In this study, we assessed pro-BNP as a useful marker for not only predicting cardiovascular outcomes as cited by many previous studies but also the mortality in postoperative patients. Not only as a cardiovascular marker, but elevated values of pro-BNP also signify volume overload while our patients only had 6.8% preoperative cardiac comorbidities, yet 27.2% of them developed postoperative cardiac complications. This factor further enhances the role of pro-BNP as not only a predictor of cardiovascular outcomes but also an independent marker of mortality with or without cardiac event. We recommend including pro-BNP as a regular preoperative assessment modality for predicting the short-term outcome of postoperative mortality as suggested by our results.
